# Plasma neurofilament light chain mediates the effect of subsyndromal symptomatic depression on cognitive decline in older adults

**DOI:** 10.3389/fnagi.2025.1547394

**Published:** 2025-05-14

**Authors:** Chunhua Zhang, Bingyu Li, Kok Pin Ng, Guojuan Huang, Xijin Wang, Min Kong, Maowen Ba

**Affiliations:** ^1^Department of Neurology, Affiliated Yantai Yuhuangding Hospital of Qingdao University, Yantai, China; ^2^Department of Neurology, Jiaozhou Branch of Shanghai East Hospital, Tongji University, Jiaozhou, China; ^3^Department of Neurology, Shanghai Tongji Hospital, School of Medicine, Tongji University, Shanghai, China; ^4^Department of Neurology, National Neuroscience Institute, Singapore, Singapore; ^5^Duke-NUS Medical School, Singapore, Singapore; ^6^Department of Neurology, Yantaishan Hospital, Yantai, China; ^7^Shandong Provincial Key Laboratory of Neuroimmune Interaction and Regulation, Yantai, China

**Keywords:** subsyndromal symptomatic depression, cognition, neurofilament light chain, mediation analysis, Alzheimer’s disease

## Abstract

**Objective:**

Subsyndromal symptomatic depression (SSD) is associated with an increased risk of cognitive impairment in non-demented older adults. However, the mechanism underlying this relationship remains unclear. This study aimed to investigate whether plasma neurofilament light chain (NfL) mediates the relationship between SSD and cognitive decline.

**Materials and methods:**

Data of 707 non-demented older adults from the Alzheimer’s Disease Neuroimaging Initiative (ADNI) cohort were analyzed. Geriatric Depression Scale (GDS) scores were collected at baseline, while plasma NfL levels and cognitive assessments were obtained at baseline, 1-year, and 2-year follow-up visits. SSD was defined as a GDS score of 1–5. Mediation analyses were performed to examine whether the rate of change in plasma NfL levels mediated the relationship between SSD and cognitive decline.

**Results:**

Participants with SSD exhibited a greater increase in plasma NfL levels and more pronounced declines in global cognition, memory, executive function, language, and processing speed over 2 years compared to non-SSD participants. The rate of change in plasma NfL levels significantly mediated the relationship between SSD and accelerated cognitive decline, particularly in global cognition, memory, language, and processing speed.

**Conclusion:**

Plasma NfL, which is related to neuroaxonal damage, may partially mediate the association between SSD and accelerated cognitive decline in non-demented older adults. These findings suggest that dynamic changes in plasma NfL levels may reflect early neurobiological alterations associated with SSD and could help identify individuals at increased risk of cognitive deterioration over a 2-year period.

## 1 Introduction

Depression is a common psychiatric disorder that affects millions of individuals worldwide (COVID-19 Mental Disorders Collaborators, 2021). In older adults, depression is associated with numerous adverse outcomes, including reduced quality of life, physical comorbidities, premature mortality, and cognitive impairment (Zis et al., 2017; Avasthi and Grover, 2018). Evidence from a large population-based cohort study, which included both Caucasian and non-Caucasian participants, shows that late-life onset of depressive symptoms is associated with a 70% higher risk of dementia and a two-fold increase risk of Alzheimer’s disease (AD) (Barnes et al., 2012).

Depressive symptoms can manifest in a spectrum of clinical syndromes, ranging from subsyndromal symptomatic depression (SSD) to major depressive disorder (MDD). SSD represents individuals who experience depressive symptoms but do not fully meet the diagnostic criteria for either major or minor depression outlined in the Diagnostic and Statistical Manual of Mental Disorders (Lyness et al., 2009). On the other hand, MDD is characterized by persistent low mood, diminished interest in activities, and significant impairment in social and occupational functioning (Uher et al., 2014). SSD is clinically important, as it is prevalent among older adults (Horwath et al., 1992; Cuijpers and Smit, 2004), significantly increases the risk of progression to MDD (Cuijpers et al., 2004) and impairs the quality of life of older adults (Rapaport and Judd, 1998; da Silva Lima and de Almeida Fleck, 2007). Furthermore, studies have shown that SSD is a major contributor to cognitive decline among older adults (Jing et al., 2024). Early identification and intervention for SSD may prevent progression to MDD (Judd et al., 1998) and mitigate associated cognitive decline (Zhang et al., 2020). Longitudinal studies further reveal that baseline SSD is associated with accelerated cognitive decline across multiple domains, including global cognition, language, executive function, and processing speed, over a 2-year period (Lee et al., 2012). Notably, individuals with persistent depressive symptoms demonstrated a higher propensity to convert to AD and exhibited significant cognitive deterioration, highlighting SSD as a potential marker for AD progression (Lee et al., 2012). Therefore, SSD represents an important window for early diagnosis and timely intervention to prevent cognitive decline before MDD sets in.

Emerging evidence suggests that depressive symptoms, including SSD, are linked to cytoarchitectural changes and neuronal injury, which may play a role in cognitive impairment (Csabai et al., 2018; Holmes et al., 2019; Williams et al., 2019; Banasr et al., 2021). However, the role of neuronal injury in the relationship between SSD and cognitive impairment in older adults remains poorly understood. Neurofilament light chain (NfL), a neuron-specific component of the axonal cytoskeleton, has been validated as a peripheral biomarker for neuroaxonal damage (Disanto et al., 2017). Elevated blood NfL levels have been reported in neurological conditions such as traumatic brain injury and AD (Disanto et al., 2017; Gaetani et al., 2019). Experimental animal models have also demonstrated associations between depression and altered neurofilament levels (Reinés et al., 2004; Cereseto et al., 2006). Hence, plasma NfL is increasingly utilized in human studies exploring depressive symptoms and neuroaxonal damage. Case–control studies show that patients with major depression exhibit significantly higher NfL levels (Bavato et al., 2021). NfL levels are highly sensitive indicators of subclinical neurodegeneration, with elevations often detectable years before the onset of clinical symptoms in both early- and late-onset AD (Preische et al., 2019; de Wolf et al., 2020). Studies in cognitively healthy older adults have found that increased NfL levels are associated with cognitive impairment (Chatterjee et al., 2018). Given the established role of NfL as a marker of neurodegeneration, investigating its role in SSD-related cognitive decline is crucial.

Previous studies have demonstrated associations between SSD and structural brain changes observed on magnetic resonance imaging, particularly in regions vulnerable to neurodegeneration, as well as cognitive decline (Jing et al., 2024). However, these findings are largely based on cross-sectional analyses, which limit causal interpretations (Jing et al., 2024). Moreover, although NfL levels have been linked to cognitive impairment in MDD (Chen et al., 2022), the mediating role of NfL in the longitudinal relationship between SSD and cognitive decline in older adults has not yet been investigated.

To address this gap, this longitudinal study of non-demented older adults, stratified into SSD and non-SSD groups, aims to investigate the relationships between depressive symptoms, plasma NfL levels, and cognitive decline over a 2-year period. We hypothesize that plasma NfL levels will mediate the association between SSD and cognitive decline among non-demented older adults.

## 2 Materials and methods

### 2.1 Alzheimer’s Disease Neuroimaging Initiative

The data used in this study were obtained from the Alzheimer’s Disease Neuroimaging Initiative (ADNI) database (http://adni.loni.usc.edu) in January 2024. ADNI is a longitudinal, multicenter study designed to characterize clinical, genetic, imaging, and biochemical biomarkers for early detection and tracking of AD progression (Weiner et al., 2015). Additional information about ADNI is available at http://www.adni-info.org. The study was conducted with institutional review board approval at each participating site. Written informed consent was obtained from all participants or their authorized representatives.

### 2.2 Participants

Non-demented participants for this study were recruited from ADNI-1, ADNI-2, and ADNI-GO who were classified as cognitively normal (CN) and mild cognitive impairment (MCI). We selected participants who had available baseline Geriatric Depression Scale (GDS) scores, longitudinal plasma NfL levels, and neuropsychological assessments conducted over a 2-year period. Exclusion criteria included: (1) missing sociodemographic data (age, gender, years of education, APOE ε4 status); (2) a diagnosis of psychiatric or neurological conditions other than AD; and (3) the presence of major depression or significant depressive symptoms (GDS score > 5). Diagnostic criteria for CN and MCI in the ADNI cohort have been previously described (Petersen et al., 2010).

### 2.3 Depression scale measurement

The 15-item GDS was used to assess depressive symptoms in the ADNI study. The total GDS scores range from 0 to 15, with higher scores indicating more severe depressive symptoms. A score of 6 or higher on the GDS is considered clinically significant for depression (Yesavage et al., 1982; Marc et al., 2008). In line with prior studies, participants with SSD were classified as having a baseline GDS score between 1 and 5, while non-SSD participants were defined as having a score of 0 (Bertens et al., 2017).

### 2.4 Apolipoprotein E genotyping

Apolipoprotein E (APOE) genotyping was performed on DNA extracted from 3 mL of blood treated with ethylenediaminetetraacetic acid in accordance with protocols provided by Cogenics (https://adni.loni.usc.edu/data-samples/adni-data/genetics-related-omics/). Participants carrying one or more copies of the ε4 allele (ε4/ε4, ε4/ε3, or ε4/ε2) were classified as APOE ε4 carriers, while those with no ε4 alleles were classified as APOE ε4 non-carriers.

### 2.5 Plasma NfL data

Plasma NfL levels were measured using the Single Molecule Array technique, which employs monoclonal antibodies and purified bovine NfL as a calibrator. All samples were analyzed in duplicate, with an analytical sensitivity of <1.0 pg./mL. Plasma NfL data from baseline, 1-year follow-up, and 2-year follow-up were included in this study. Further methodological details are available at http://adni.loni.usc.edu.

### 2.6 Cognitive assessments

ADNI participants undergo a wide spectrum of clinical and cognitive assessments (Aisen et al., 2010). In this study, global cognition was evaluated using the Alzheimer’s Disease Assessment Scale–Cognitive Subscale, which includes both the 11-item (ADAS-Cog 11) and 13-item versions (ADAS-Cog 13) (Mohs et al., 1997). Memory function was assessed using the ADNI Memory Composite (ADNI-MEM) (Crane et al., 2012). Executive function was measured using the ADNI Executive Function Composite (ADNI-EF) (Gibbons et al., 2012). Psychomotor processing speed and attention was evaluated with the Trail Making Test Part A (TMT-A) and Trail Making Test Part B (TMT-B). Language ability was assessed using the ADNI Language Composite (ADNI-LAN) (Choi et al., 2020). Cognitive data from baseline, 1-year follow-up, and 2-year follow-up were included in this study. Further detailed information is available at https://adni.loni.usc.edu/methods/.

### 2.7 Statistical analysis

All statistical analyses were conducted using R software (version 4.3.1, The R Foundation for Statistical Computing). Statistical significance was set at a two-tailed *p*-value of < 0.05.

Continuous variables were presented as means with standard deviations, and categorical variables were presented as frequencies and percentages. Independent t-tests were used to compare continuous variables, and chi-square (χ^2^) tests were used for categorical variables. When assumptions of normality or homogeneity of variance were not met, the Wilcoxon rank-sum test was used. Analysis of covariance was performed to evaluate baseline cognitive outcomes, adjusting for potential confounders including age, gender, years of education, diagnostic status (CN vs. MCI), and APOE ε4 status.

The rate of change in cognitive measures and plasma NfL levels was calculated using linear mixed-effects models, as previously established (Preische et al., 2019; Rubinski et al., 2024). This model estimated longitudinal changes by including time from baseline as the independent variable, with random slopes and intercepts to account for individual variability.

Before conducting the analysis, outliers in *Δ* plasma NfL levels were identified using the inter-quartile range (IQR) method. Data points below Q1–1.5 × IQR or above Q3 + 1.5 × IQR were classified as outliers and excluded from further analysis. Outliers were excluded to minimize the influence of extreme values and enhance the statistical robustness of the analysis.

Multiple linear regression models were used to examine the relationship between SSD, longitudinal plasma NfL levels, and longitudinal cognitive measures. All models were adjusted for age, gender, years of education, diagnostic status (CN vs. MCI), and APOE ε4 status. Baseline MMSE scores were included as a covariate in the longitudinal cognitive measures analyses to control for differences in baseline cognitive status between the CN and MCI groups. Region-wise multiple comparisons were corrected using the Benjamini-Hochberg false discovery rate (FDR) method (FDR-corrected *p* < 0.05 for 7 cognitive measures).

Mediation analyses were conducted to examine the relationships among SSD, the rate of change in plasma NfL levels, and cognitive decline, using the “BruceR” package (R version 4.3.1, https://psychbruce.github.io/bruceR/). In this model, SSD was specified as the independent variable (X), the rate of change in cognitive function as the dependent variable (Y), and the rate of change in plasma NfL levels as the mediator (M). The mediation effect was considered present if the following conditions were met: (1) SSD was significantly associated with the rate of change in plasma NfL levels; (2) SSD was significantly associated with the rate of change in cognitive function; and (3) the rate of change in plasma NfL levels was significantly associated with the rate of change in cognitive function. The indirect (mediated) effect was estimated using 1,000 bootstrapped iterations, with all paths adjusted for the aforementioned covariates. The proportion of the mediation effect was calculated by dividing the indirect effect by the total effect.

## 3 Results

### 3.1 Participant characteristics

Among 707 non-demented older adults, 467 participants were classified as SSD (mean age: 71.17 years, SD: 6.98), while 240 were classified as non-SSD (mean age: 72.98 years, SD: 6.48). The distribution of CN and MCI participants (*p* < 0.001) and age (*p* < 0.001) were significantly different between the SSD and non-SSD groups, with a higher proportion of MCI participants and younger age in the SSD group compared to the non-SSD group. However, there were no statistically significant differences in the gender distribution, years of education, APOE ε4 carrier status, and baseline plasma NfL levels between the SSD and non-SSD groups (*p* > 0.05; Table 1).

**Table 1:** The demographic characteristics of participants.

	Non-SSD (*N* = 240)	SSD (*N* = 467)	*P*
CN/MCI	143/97 (60%/40%)	134/333 (29%/71%)	<0.001
Age, years	72.98 (6.48)	71.17 (6.98)	<0.001
Female, *n* (%)	114 (48%)	229 (49%)	0.76
Year of education	16.41 (2.64)	16.36 (2.60)	0.9
APOE ε4 carrier, *n* (%)	96 (40%)	197 (42%)	0.63
Plasma NfL, pg./mL	34.52 (14.43)	34.45 (14.85)	0.87

Data are presented as mean (SD). SSD, subsyndromal symptomatic depression; CN, cognitively normal; MCI, mild cognitive impairment; APOE, apolipoprotein E; NfL, neurofilament light. Statistical significance was defined as *P* < 0.05.

### 3.2 SSD and plasma NfL

**Figure 1:**
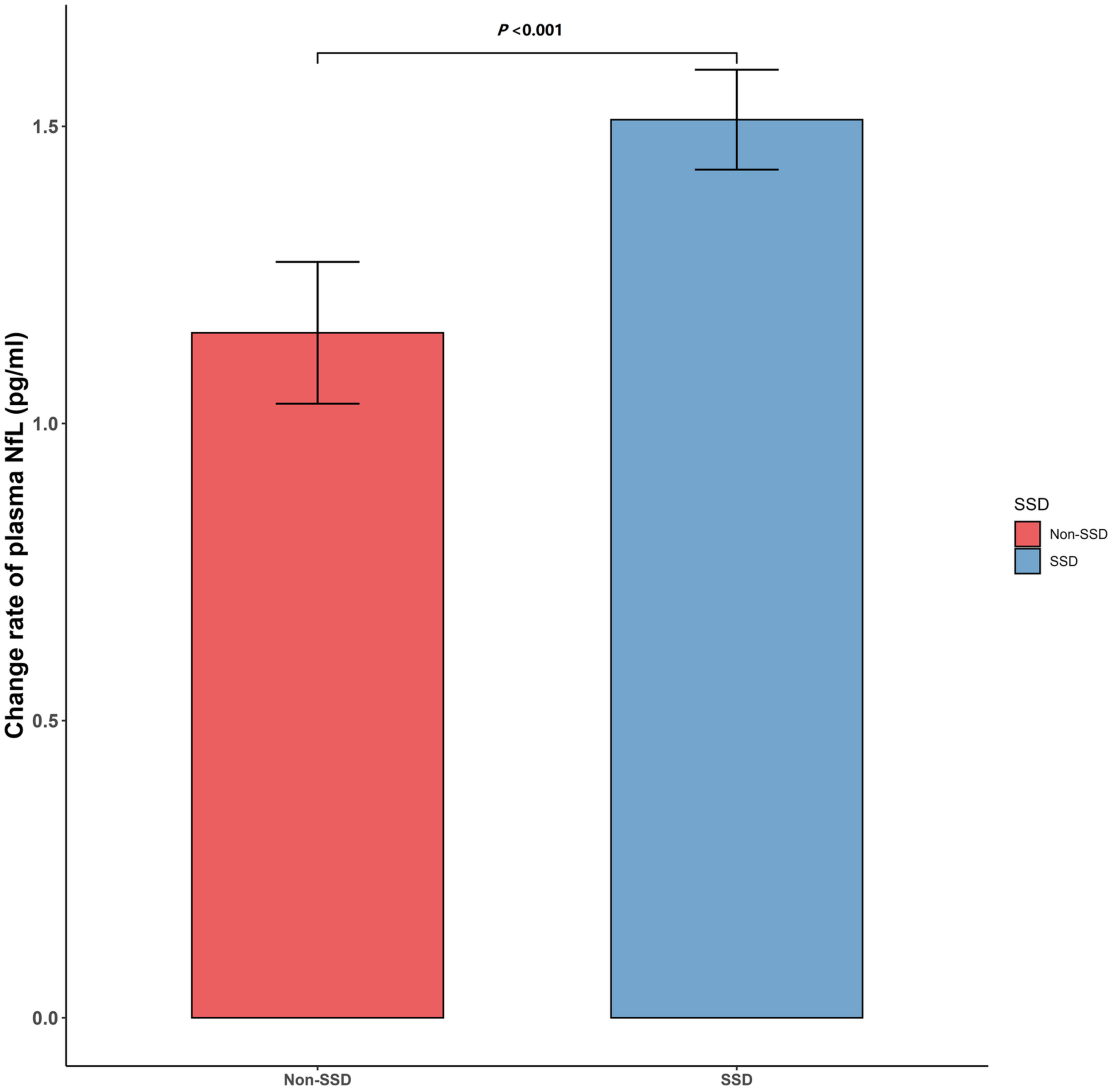
Associations between SSD and plasma NfL. *p*-values were adjusted for age, gender, years of education, diagnostic status (CN vs. MCI), and APOE ε4 status. *Δ* represents the rate of change in measurements. Statistical significance was set at *p* < 0.05. CN, cognitively normal; MCI, mild cognitive impairment; SSD, subsyndromal symptomatic depression; APOE, apolipoprotein E; NfL, neurofilament light.

Linear regression model was applied to examine the associations between SSD and rate of change in plasma NfL levels, adjusting for age, gender, years of education, diagnostic status, and APOE ε4 status. We found that SSD participants experienced a more rapid increase in plasma NfL levels compared to non-SSD participants (see Figure 1).

### 3.3 SSD and cognitive performance

Baseline cognitive performance and the rate of change in cognitive function (*Δ* cognition) for SSD and non-SSD participants are summarized in Table 2.

**Table 2:** Cognitive function test results at baseline and after follow-up.

Outcome	Non-SSD (*N* = 240)	SSD (*N* = 467)	*P*	FDR-corrected *P*
Baseline cognitive measures				
ADAS-Cog 11	6.90 (3.65)	8.16 (4.32)	0.06	0.08
ADAS-Cog 13	10.92 (5.87)	13.08 (6.65)	0.02	0.05
ADNI-MEM	0.92 (0.83)	0.65 (0.82)	0.03	0.06
ADNI-EF	0.77 (0.91)	0.53 (0.82)	0.00	0.01
TMT-A	35.38 (14.30)	36.83 (14.73)	0.32	0.32
TMT-B	90.50 (51.70)	98.66 (53.10)	0.13	0.15
ADNI-LAN	0.76 (0.77)	0.54 (0.75)	0.01	0.04
Change rate of cognitive measures				
Δ ADAS-Cog 11	0.02 (0.92)	0.42 (1.18)	0.02	0.03
Δ ADAS-Cog 13	0.00 (1.16)	0.53 (1.48)	0.01	0.02
Δ ADNI-MEM	0.02 (0.14)	−0.03 (0.16)	0.00	0.02
Δ ADNI-EF	−0.01 (0.12)	−0.03 (0.13)	0.07	0.08
Δ TMT-A	0.35 (1.82)	0.83 (2.43)	0.03	0.05
Δ TMT-B	3.04 (7.04)	4.09 (8.01)	0.49	0.49
Δ ADNI-LAN	−0.01 (0.13)	−0.05 (0.15)	0.02	0.03

Data are presented as mean (SD). *P*-values were calculated adjusting for age, gender, years of education, diagnostic status (CN vs. MCI), and APOE ε4 status. Baseline MMSE scores were included as a covariate in the longitudinal cognitive measures analyses. Region-wise multiple comparisons were corrected using the Benjamini-Hochberg false discovery rate (FDR) method (FDR-corrected *P* < 0.05 for 7 cognitive measures). Δ represents the rate of change in measurements. SSD, Subsyndromal Symptomatic Depression; ADAS-Cog11/13, Alzheimer’s Disease Assessment Scale-11/13 item subscale; ADNI-MEM, ADNI Memory Composite; ADNI-EF, ADNI Executive Function Composite; ADNI-LAN, ADNI Language Composite; TMT-A/B, Trail Making Test Part A/B.

At baseline, participants in the SSD group exhibited significantly higher ADAS-Cog 13 scores (FDR-corrected *p* = 0.05), lower ADNI-EF (FDR-corrected *p* = 0.01) and lower ADNI-LAN (FDR-corrected *p* = 0.04) scores compared to non-SSD participants. These findings suggest that individuals with SSD had greater impairments in global cognition, executive function, and language abilities at baseline. A trend toward lower memory performance (measured by ADNI-MEM, FDR-corrected *p* = 0.06) was also observed in the SSD group. No significant between-group differences were observed for ADAS-Cog 11, TMT-A, or TMT-B (FDR-corrected *p* > 0.05).

During the 2-year follow-up, the SSD group exhibited significantly greater declines in global cognition (assessed by ADAS-Cog 11 and ADAS-Cog 13), memory (ADNI-MEM), processing speed (TMT-A), and language function (ADNI-LAN), compared to the non-SSD group (all FDR-corrected *p* < 0.05). Although the SSD group showed a trend toward greater decline in executive function (ADNI-EF), the association was not statistically significant after FDR correction (FDR-corrected *p* = 0.08). No significant differences were observed in the change rate of TMT-B (FDR-corrected *p* > 0.05).

All analyses were adjusted for age, gender, years of education, diagnostic status (CN vs. MCI), and APOE ε4 status. Baseline MMSE scores were included as a covariate in the longitudinal cognitive measures analyses.

### 3.4 Associations between plasma NfL and cognitive performance

**Figure 2:**
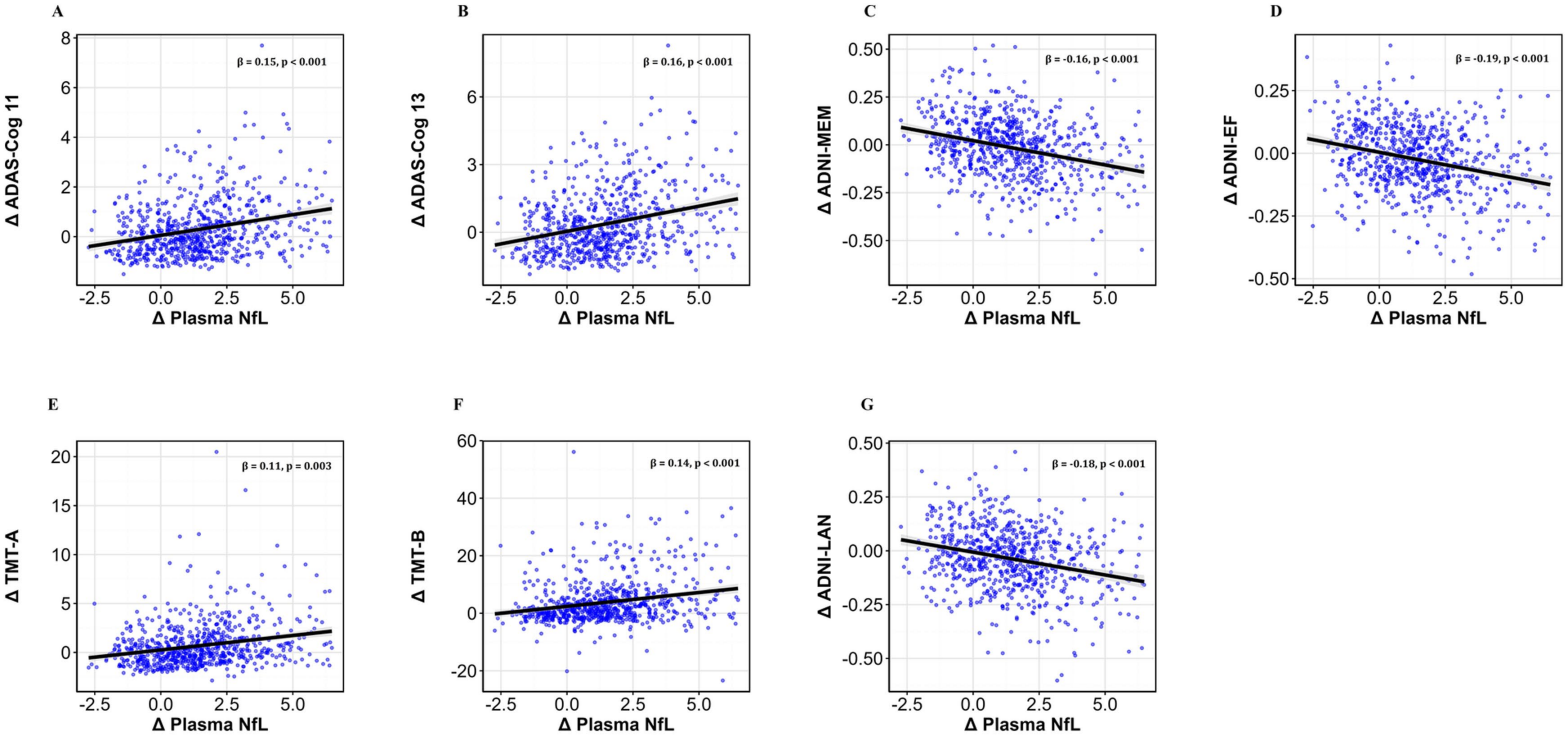
Associations between plasma NfL levels and cognitive performance. Scatterplots show the linear regression line (solid black) and 95% confidence interval (shaded area) depicting the associations between the 2-year rate of change in plasma NfL levels (Δ Plasma NfL) and the corresponding rate of change in cognitive performance, including: **(A)** ADAS-Cog 11, **(B)** ADAS-Cog 13, **(C)** ADNI-MEM, **(D)** ADNI-EF, **(E)** TMT-A, **(F)** TMT-B, and **(G)** ADNI-LAN. Standardized β-values and *p*-values are shown in each panel. All models were adjusted for age, gender, years of education, diagnostic status (CN vs. MCI), APOE ε4 status, and baseline MMSE scores. Δ represents the rate of change in measurements. Statistical significance was defined as *p* < 0.05. CN, cognitively normal; MCI, mild cognitive impairment; APOE, apolipoprotein E; NfL, neurofilament light; ADAS-Cog 11/13, Alzheimer’s Disease Assessment Scale 11−/13-item version; ADNI-MEM, ADNI memory composite; ADNI-EF, ADNI executive function composite; ADNI-LAN, ADNI language composite; TMT-A/B, Trail Making Test Part A/B.

Over the 2-year follow-up, greater increases in plasma NfL levels were significantly associated with steeper declines across multiple cognitive domains (Figure 2). Specifically, higher NfL changes were related to worsening global cognition (Figure 2A: ADAS-Cog 11: *β* = 0.15, *p* < 0.001; Figure 2B: ADAS-Cog 13: β = 0.16, *p* < 0.001), memory (Figure 2C: ADNI-MEM: β = −0.16, *p* < 0.001), executive function (Figure 2D: ADNI-EF: β = −0.19, *p* < 0.001), language abilities (Figure 2G: ADNI-LAN: β = −0.18, *p* < 0.001), as well as processing speed and attention (Figure 2E: TMT-A: β = 0.11, *p* = 0.003; Figure 2F: TMT-B: β = 0.14, *p* < 0.001). All models were adjusted for age, gender, education, diagnostic status, baseline MMSE, and APOE ε4 status.

### 3.5 Mediation effect of the rate of change in plasma NfL levels in the association between SSD and cognitive decline

**Figure 3:**
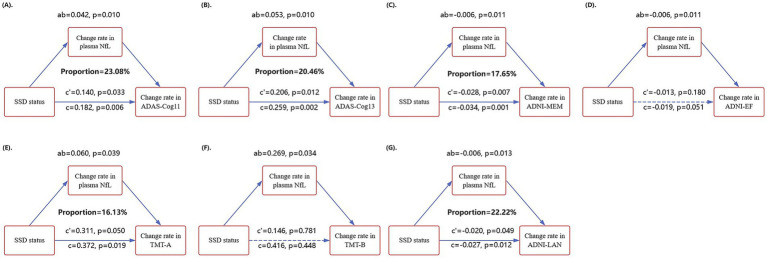
Mediation analysis diagram illustrating the relationship between SSD and cognitive performance. In the mediation analysis, the independent variable (X) was SSD status, the mediator (M) was the 2-year rate of change in plasma NfL levels, and the dependent variable (Y) was the rate of change in specific cognitive measures: **(A)** ADAS-Cog 11, **(B)** ADAS-Cog 13, **(C)** ADNI-MEM, **(D)** ADNI-EF, **(E)** TMT-A, **(F)** TMT-B, and **(G)** ADNI-LAN. Δ represents the rate of change in measurements. The indirect effect (ab), direct effect (c′), and total effect (c) are reported. All models were adjusted for age, gender, years of education, diagnostic status (CN vs. MCI), APOE ε4 status, and baseline MMSE scores. SSD, subsyndromal symptomatic depression; CN, cognitively normal; MCI, mild cognitive impairment; APOE, apolipoprotein E; NfL, neurofilament light; ADAS-Cog11/13, Alzheimer’s Disease Assessment Scale-11/13 item subscale; ADNI-MEM, ADNI Memory Composite; ADNI-EF, ADNI Executive Function Composite; ADNI-LAN, ADNI Language Composite; TMT-A/B, Trail Making Test Part A/B.

As shown in Figure 3, mediation analyses revealed that the 2-year rate of change in plasma NfL significantly mediated the association between SSD and cognitive decline across several cognitive domains. Specifically, plasma NfL partially mediated the relationship between SSD and global cognition, as assessed by ADAS-Cog 11 (Figure 3A: ab = 0.042, *p* = 0.010; mediation proportion = 23.08%) and ADAS-Cog 13 (Figure 3B: ab = 0.053, *p* = 0.010; 20.46%). Similar effects were observed for memory (Figure 3C: ADNI-MEM: ab = −0.006, *p* = 0.011; 17.65%), language (Figure 3G: ADNI-LAN: ab = −0.006, *p* = 0.013; 22.22%), and processing speed (Figure 3E: TMT-A: ab = 0.060, *p* = 0.039; 16.13%). In contrast, no mediation effect was found for executive function (Figure 3D: ADNI-EF), as neither the total nor direct effects reached statistical significance. All models were adjusted for age, sex, years of education, diagnostic status (CN vs. MCI), APOE ε4 status, and baseline MMSE score.

## 4 Discussion

Our study demonstrates that SSD in non-demented older adults is significantly associated with a greater increase in plasma NfL levels and more pronounced declines in both global and domain-specific cognitive performance over the 2-year follow-up period. Furthermore, the relationship between SSD and cognitive decline was partially mediated by the change rate in plasma NfL, with significant mediation effects observed in global cognition, memory, language, and processing speed. These findings offer important insights into mechanisms linking SSD, neuroaxonal damage, and cognitive deterioration. Our results suggest that SSD may accelerate neuroaxonal damage, as reflected by elevated plasma NfL levels, contributing to faster cognitive deterioration.

The elevated NfL levels observed in the SSD group align with previous neuroimaging studies that have shown reduced gray matter volume in the hippocampus and decreased white matter integrity in the fornix, posterior cingulum, and corpus callosum (Zhang et al., 2020; Touron et al., 2022). These neuroanatomical changes may help explain the association between SSD and cognitive function. However, the mediating role of NfL in this association remains underexplored, with only one cross-sectional study having examined the mediation of the association between depressive symptoms and cognitive function by NfL levels (Xu et al., 2024). Our study addresses this gap by demonstrating that plasma NfL levels mediate the relationship between SSD and cognitive decline over time, with significant effects observed across multiple cognitive domains, including global cognition, memory, language and processing speed.

The precise biological mechanisms linking SSD to cognitive decline through NfL remain not fully understood, though several plausible pathways have been suggested. Depression is known to induce neurobiological changes, such as neuroinflammation, oxidative stress, and dysregulation of the hypothalamic–pituitary–adrenal axis, all of which have been associated with neuroaxonal damage (Kita et al., 2000; Maes et al., 2009; Kaster et al., 2012; Howes and McCutcheon, 2017; Rodrigues-Amorim et al., 2020). These pathological processes may accelerate cognitive decline by impairing key brain regions involved in memory, executive function, and other cognitive functions. While most prior research has primarily focused on these mechanisms in MDD, our study expands this understanding by demonstrating that elevated plasma NfL levels, which have been linked to cognitive decline in neurodegenerative and psychiatric populations (Bavato et al., 2024), are also observed in individuals with SSD. We propose that neuroaxonal injury, as indicated by increased plasma NfL levels, begins early in SSD and contributes to cognitive decline. This perspective expands the current understanding, which has focused largely on MDD, and highlights neuroaxonal damage as a potential early biomarker for cognitive impairment in SSD.

Our findings suggest that elevated plasma NfL levels in individuals with SSD may reflect underlying neuroaxonal injury that contributes to cognitive decline. Given the established association between NfL and neuroaxonal damage, this pattern may represent one possible biological pathway linking subthreshold depressive symptoms and early neurodegenerative processes. Our findings highlight the relevance of plasma NfL dynamics in characterizing early neurobiological alterations in non-demented older adults with SSD.

To our knowledge, this is the first study to demonstrate that plasma NfL levels mediate the association between SSD and cognitive decline, building on prior research that has predominantly focused on MDD (Chen et al., 2022). Unlike previous studies, which typically relied on a single cognitive measure (Chen et al., 2022), our research provides a comprehensive evaluation across multiple cognitive domains, including attention, processing speed, memory, language, and executive function. Moreover, by focusing on participants with minimal to mild depressive symptoms, which are common in older adults, this study offers valuable insights into the impact of these symptoms on cognitive decline, a critical area for early intervention.

Several limitations of this study should be acknowledged. First, the data were retrospectively obtained from the ADNI cohort, which may have introduced selection bias. The ADNI sample primarily consisted of non-Hispanic White participants with relatively high levels of education and socioeconomic status. Second, the study focused on baseline depressive symptoms and their association with cognitive decline over a 2-year period, without accounting for potential fluctuations in depressive symptom severity during follow-up. Participants whose depressive symptoms may have changed throughout the study period were not excluded, as the primary objective was to examine the influence of baseline symptoms on cognitive outcomes and the mediating role of plasma NfL. Third, critical factors such as cardiovascular risk, renal function, and physical activity, which are known to influence NfL levels, were not systematically assessed in our sample (Barro et al., 2020). Fourth, the inclusion of both CN and MCI participants may have introduced residual heterogeneity. To account for group differences and baseline cognitive function, diagnostic status (CN vs. MCI) and baseline MMSE were included as covariates in all statistical models. Moreover, unmeasured AD-related pathologies, such as amyloid-*β* or phosphorylated tau burden, may have influenced cognitive trajectories and biomarker dynamics, particularly in the MCI subgroup. Future studies incorporating AD biomarkers and more diagnostically balanced samples are needed to clarify diagnosis-specific associations and underlying pathophysiological mechanisms.

Our findings indicate that the change in plasma NfL may partially mediate the relationship between SSD and accelerated decline in global cognition, memory, language, and processing speed. These results suggest that plasma NfL dynamics may serve as an informative biological indicator for identifying non-demented older adults with SSD who are at increased risk of cognitive decline over a 2-year period.

## Data Availability

The original contributions presented in the study are included in the article/supplementary material, further inquiries can be directed to the corresponding authors.

## Group member of Alzheimer’s Disease Neuroimaging Initiative

Data used in the preparation of this article were obtained from the Alzheimer's Disease Neuroimaging Initiative (ADNI) database (http://adni.loni.usc.edu). As such, the investigators within the ADNI contributed to the design and implementation of ADNI and/or provided data but did not participate in the analysis or writing of this report. A complete listing of ADNI investigators can be found at: https://adni.loni.usc.edu/wp-content/uploads/how_to_apply/ADNI_Acknowledgement_List.pdf.
